# Supplementary material for: Psychometric properties of measures of upper limb activity performance in adults with and without spasticity undergoing neurorehabilitation–A systematic review
Source: PLoS One. 2021 Feb 11;16(2):e0246288. doi: 10.1371/journal.pone.0246288 (PMC7877653; doi:10.1371/journal.pone.0246288)
Supplement: S1 File — MEDLINE search strategy and terms used in search. (DOCX) [file pone.0246288.s002.docx]

**S1 File. Search Strategy and Terms**

**Medline**

Filters

- Limits: published in English

(“title of assessment tool”) AND hasabstract AND (instrumentation[sh] OR methods[sh] OR Validation Studies[pt] OR Comparative Study[pt] OR "psychometrics"[MeSH] OR psychometr*[tiab] OR clinimetr*[tw] OR clinometr*[tw] OR "outcome assessment (health care)"[MeSH] OR outcome assessment[tiab] OR outcome measure*[tw] OR "observer variation"[MeSH] OR observer variation[tiab] OR "Health Status Indicators"[Mesh] OR "reproducibility of results"[MeSH] OR reproducib*[tiab] OR "discriminant analysis"[MeSH] OR reliab*[tiab] OR unreliab*[tiab] OR valid*[tiab] OR coefficient[tiab] OR homogeneity[tiab] OR homogeneous[tiab] OR "internal consistency"[tiab] OR (cronbach*[tiab] AND (alpha[tiab] OR alphas[tiab])) OR (item[tiab] AND (correlation*[tiab] OR selection*[tiab] OR reduction*[tiab])) OR agreement[tiab] OR precision[tiab] OR imprecision[tiab] OR "precise values"[tiab] OR test-retest[tiab] OR (test[tiab] AND retest[tiab]) OR (reliab*[tiab] AND (test[tiab] OR retest[tiab])) OR stability[tiab] OR interrater[tiab] OR inter-rater[tiab] OR intrarater[tiab] OR intra-rater[tiab] OR intertester[tiab] OR inter-tester[tiab] OR intratester[tiab] OR intra-tester[tiab] OR interobserver[tiab] OR inter-observer[tiab] OR intraobserver[tiab] OR intra-observer[tiab] OR intertechnician[tiab] OR inter-technician[tiab] OR intratechnician[tiab] OR intra-technician[tiab] OR interexaminer[tiab] OR inter-examiner[tiab] OR intraexaminer[tiab] OR intra-examiner[tiab] OR interassay[tiab] OR inter-assay[tiab] OR intraassay[tiab] OR intra-assay[tiab] OR interindividual[tiab] OR inter-individual[tiab] OR intraindividual[tiab] OR intra-individual[tiab] OR interparticipant[tiab] OR inter-participant[tiab] OR intraparticipant[tiab] OR intra-participant[tiab] OR kappa[tiab] OR kappa's[tiab] OR kappas[tiab] OR repeatab*[tiab] OR ((replicab*[tiab] OR repeated[tiab]) AND (measure[tiab] OR measures[tiab] OR findings[tiab] OR result[tiab] OR results[tiab] OR test[tiab] OR tests[tiab])) OR generaliza*[tiab] OR generalisa*[tiab] OR concordance[tiab] OR (intraclass[tiab] AND correlation*[tiab]) OR discriminative[tiab] OR "known group"[tiab] OR factor analysis[tiab] OR factor analyses[tiab] OR dimension*[tiab] OR subscale*[tiab] OR (multitrait[tiab] AND scaling[tiab] AND (analysis[tiab] OR analyses[tiab])) OR item discriminant[tiab] OR interscale correlation*[tiab] OR error[tiab] OR errors[tiab] OR "individual variability"[tiab] OR (variability[tiab] AND (analysis[tiab] OR values[tiab])) OR (uncertainty[tiab] AND (measurement[tiab] OR measuring[tiab])) OR "standard error of measurement"[tiab] OR sensitiv*[tiab] OR responsive*[tiab] OR ((minimal[tiab] OR minimally[tiab] OR clinical[tiab] OR clinically[tiab]) AND (important[tiab] OR significant[tiab] OR detectable[tiab]) AND (change[tiab] OR difference[tiab])) OR (small*[tiab] AND (real[tiab] OR detectable[tiab]) AND (change[tiab] OR difference[tiab])) OR meaningful change[tiab] OR "ceiling effect"[tiab] OR "floor effect"[tiab] OR "Item response model"[tiab] OR IRT[tiab] OR Rasch[tiab] OR "Differential item functioning"[tiab] OR DIF[tiab] OR "computer adaptive testing"[tiab] OR "item bank"[tiab] OR "cross-cultural equivalence"[tiab])) NOT (("addresses"[Publication Type] OR "biography"[Publication Type] OR "case reports"[Publication Type] OR "comment"[Publication Type] OR "directory"[Publication Type] OR "editorial"[Publication Type] OR "festschrift"[Publication Type] OR "interview"[Publication Type] OR "lectures"[Publication Type] OR "legal cases"[Publication Type] OR "legislation"[Publication Type] OR "letter"[Publication Type] OR "news"[Publication Type] OR "newspaper article"[Publication Type] OR "patient education handout"[Publication Type] OR "popular works"[Publication Type] OR "congresses"[Publication Type] OR "consensus development conference"[Publication Type] OR "consensus development conference, nih"[Publication Type] OR "practice guideline"[Publication Type]) OR ("animals"[MeSH Terms] NOT "humans"[MeSH Terms])

“Assessment tool” (Replace assessment tool with one below)

- “Action Research Arm Test” OR “ARAT” = Y
- “Arm Activity Measure” OR “ArMA” = Y
- “Assessment of Quality of Life” OR “AQoL” = Y
- “Barthel Index” = Y
- “Chedokee McMaster Assessment” OR “ Chedokee Assessment” OR “Chedokee McMaster Stroke Assessment” OR “CMSA” OR “CMA” = Y
- “Disability Assessment Scale” OR “DAS” = Y
- “European Quality of Life Five Dimension Scale” OR “ Euro-QoL” OR “EQ-5D” = Y
- “Frenchay Arm Test” = Y
- “Modified Frenchay Arm Test” OR “Modified Frenchay Scale” = Y
- “Functional Independence Measure” OR “FIM” = Y
- “Global Assessment Scale” OR “ GAS” = Y
- “Goal Attainment Scaling” OR “GAS” = Y
- “Goal Attainment Scaling using 10-point categorical scale for daily activities” OR “GAS using 10-point categorical scale for daily activities” = Y
- “Klein-Bell Activities of Daily Living Scale” OR “”Klein-Bell ADL Scale” = Y
- “Leeds Adult Spasticity Impact Scale” OR “LASIS” OR “Patient Disability and Carer Burden Scale” OR “8-item patient disability scale and 4-item carer burden scale” = Y
- “Medical Outcomes Study 36 items Short-Form Health Status Survey” OR “SF-36” = Y
- “Motor Activity Log” OR “Upper Extremity Motor Activity Log” OR “UE MAL” OR “MAL” = Y
- “Motor Activity Log – 28” OR “MAL-28” OR = Y
- “Motor Activity Log - 5” OR “MAL – 5” = Y
- “Modified Motor Assessment Scale” OR “MAS” = Y
- “Motricity Index” OR “MI” = Y
- “Oxford Handicap Scale” OR “OHS” = Y
- “Rivermead Motor Assessment” OR “RMA” = Y
- “Stroke Adapted Sickness Impact Profile” OR “SA-SIP30” = Y
- “Stroke Impact Scale” OR “SIS” = Y
- “9 hole peg test” OR “nine-hole peg test” OR “NHPT” = Y
