# Supplementary material for: Psychometric properties of measures of upper limb activity performance in adults with and without spasticity undergoing neurorehabilitation–A systematic review
Source: PLoS One. 2021 Feb 11;16(2):e0246288. doi: 10.1371/journal.pone.0246288 (PMC7877653; doi:10.1371/journal.pone.0246288)
Supplement: S1 Table — This file details reasons for and numbers of studies excluded. (DOCX) [file pone.0246288.s003.docx]

| **S1 Table.** **Full text exclusion reasons (PRISMA)** | | | | | |
| --- | --- | --- | --- | --- | --- |
| Outcome Measure | Exclusion reason | n = | Outcome Measure | Exclusion reason | n = |
| ARAT | Psychometric properties not evaluated | 24 | ArmA | Psychometric properties not evaluated | 2 |
|  | Conference proceedings | 0 |  | Conference proceedings | 0 |
|  | Not conducted in English | 15 |  | Not conducted in English | 0 |
|  | Modified or incorrect measure | 3 |  | Modified or incorrect measure | 0 |
|  | Not original research | 1 |  | Not original research | 0 |
|  | Protocol only | 0 |  | Protocol only | 0 |
|  | Participants outside criteria | 0 |  | Participants outside criteria | 0 |
|  |  |  |  |  |  |
| AQoL | Psychometric properties not evaluated | 2 | BI | Psychometric properties not evaluated | 56 |
|  | Conference proceedings | 0 |  | Conference proceedings | 1 |
|  | Not conducted in English | 0 |  | Not conducted in English | 23 |
|  | Modified or incorrect measure | 0 |  | Modified or incorrect measure | 15 |
|  | Not original research | 0 |  | Not original research | 5 |
|  | Protocol only | 0 |  | Protocol only | 0 |
|  | Participants outside criteria | 0 |  | Participants outside criteria | 17 |
|  |  |  |  |  |  |
| CMSA | Psychometric properties not evaluated | 14 | DAS | Psychometric properties not evaluated | 3 |
|  | Conference proceedings | 0 |  | Conference proceedings | 1 |
|  | Not conducted in English | 0 |  | Not conducted in English | 1 |
|  | Modified or incorrect measure | 6 |  | Modified or incorrect measure | 5 |
|  | Not original research | 0 |  | Not original research | 0 |
|  | Protocol only | 0 |  | Protocol only | 0 |
|  | Participants outside criteria | 1 |  | Participants outside criteria | 0 |
|  |  |  |  |  |  |
| EQ-5D | Psychometric properties not evaluated | 3 | FAT | Psychometric properties not evaluated | 1 |
|  | Conference proceedings | 0 |  | Conference proceedings | 0 |
|  | Not conducted in English | 8 |  | Not conducted in English | 0 |
|  | Modified or incorrect measure | 3 |  | Modified or incorrect measure | 1 |
|  | Not original research | 2 |  | Not original research | 0 |
|  | Protocol only | 0 |  | Protocol only | 0 |
|  | Participants outside criteria | 4 |  | Participants outside criteria | 0 |
|  |  |  |  |  |  |
| mFAT | Psychometric properties not evaluated | 6 | FIM | Psychometric properties not evaluated | 44 |
|  | Conference proceedings | 4 |  | Conference proceedings | 1 |
|  | Not conducted in English | 3 |  | Not conducted in English | 25 |
|  | Modified or incorrect measure | 4 |  | Modified or incorrect measure | 19 |
|  | Not original research | 1 |  | Not original research | 0 |
|  | Protocol only | 0 |  | Protocol only | 0 |
|  | Participants outside criteria | 0 |  | Participants outside criteria | 36 |
|  |  |  |  |  |  |
| GAS | Psychometric properties not evaluated | 20 | GAS-10 point | Psychometric properties not evaluated | 0 |
|  | Conference proceedings | 2 |  | Conference proceedings | 0 |
|  | Not conducted in English | 2 |  | Not conducted in English | 0 |
|  | Modified or incorrect measure | 2 |  | Modified or incorrect measure | 0 |
|  | Not original research | 3 |  | Not original research | 0 |
|  | Protocol only | 2 |  | Protocol only | 0 |
|  | Participants outside criteria | 9 |  | Participants outside criteria | 0 |
|  |  |  |  |  |  |
| Global Assessment Scale | Psychometric properties not evaluated | 1 | Klein Bell ADL Scale | Psychometric properties not evaluated | 3 |
|  | Conference proceedings | 0 |  | Conference proceedings | 0 |
|  | Not conducted in English | 0 |  | Not conducted in English | 3 |
|  | Modified or incorrect measure | 5 |  | Modified or incorrect measure | 2 |
|  | Not original research | 0 |  | Not original research | 1 |
|  | Protocol only | 0 |  | Protocol only | 0 |
|  | Participants outside criteria | 0 |  | Participants outside criteria | 6 |
|  |  |  |  |  |  |
| LASIS | Psychometric properties not evaluated | 2 | SF-36 | Psychometric properties not evaluated | 21 |
|  | Conference proceedings | 0 |  | Conference proceedings | 0 |
|  | Not conducted in English | 0 |  | Not conducted in English | 2 |
|  | Modified or incorrect measure | 0 |  | Modified or incorrect measure | 5 |
|  | Not original research | 0 |  | Not original research | 0 |
|  | Protocol only | 0 |  | Protocol only | 0 |
|  | Participants outside criteria | 0 |  | Participants outside criteria | 1 |
|  |  |  |  |  |  |
| MAL | Psychometric properties not evaluated | 10 | MAL-5 | Psychometric properties not evaluated | 0 |
|  | Conference proceedings | 2 |  | Conference proceedings | 0 |
|  | Not conducted in English | 6 |  | Not conducted in English | 0 |
|  | Modified or incorrect measure | 13 |  | Modified or incorrect measure | 0 |
|  | Not original research | 1 |  | Not original research | 0 |
|  | Protocol only | 0 |  | Protocol only | 0 |
|  | Participants outside criteria | 0 |  | Participants outside criteria | 0 |
|  |  |  |  |  |  |
| MAL-28 | Psychometric properties not evaluated | 0 | MI | Psychometric properties not evaluated | 19 |
|  | Conference proceedings | 0 |  | Conference proceedings | 2 |
|  | Not conducted in English | 1 |  | Not conducted in English | 3 |
|  | Modified or incorrect measure | 1 |  | Modified or incorrect measure | 5 |
|  | Not original research | 0 |  | Not original research | 0 |
|  | Protocol only | 0 |  | Protocol only | 0 |
|  | Participants outside criteria | 0 |  | Participants outside criteria | 1 |
|  |  |  |  |  |  |
| NHPT | Psychometric properties not evaluated | 51 | OHS | Psychometric properties not evaluated | 22 |
|  | Conference proceedings | 13 |  | Conference proceedings | 1 |
|  | Not conducted in English | 9 |  | Not conducted in English | 0 |
|  | Modified or incorrect measure | 0 |  | Modified or incorrect measure | 1 |
|  | Not original research | 2 |  | Not original research | 0 |
|  | Protocol only | 1 |  | Protocol only | 0 |
|  | Participants outside criteria | 4 |  | Participants outside criteria | 0 |
|  |  |  |  |  |  |
| PDC/CBS | Psychometric properties not evaluated | 0 | RMA | Psychometric properties not evaluated | 9 |
|  | Conference proceedings | 0 |  | Conference proceedings | 4 |
|  | Not conducted in English | 0 |  | Not conducted in English | 4 |
|  | Modified or incorrect measure | 0 |  | Modified or incorrect measure | 9 |
|  | Not original research | 0 |  | Not original research | 0 |
|  | Protocol only | 0 |  | Protocol only | 0 |
|  | Participants outside criteria | 0 |  | Participants outside criteria | 0 |
|  |  |  |  |  |  |
| RMA-UL | Psychometric properties not evaluated | 9 | SA-SIP | Psychometric properties not evaluated | 0 |
|  | Conference proceedings | 4 |  | Conference proceedings | 0 |
|  | Not conducted in English | 4 |  | Not conducted in English | 2 |
|  | Modified or incorrect measure | 8 |  | Modified or incorrect measure | 0 |
|  | Not original research | 0 |  | Not original research | 1 |
|  | Protocol only | 0 |  | Protocol only | 0 |
|  | Participants outside criteria | 0 |  | Participants outside criteria | 0 |
|  |  |  |  |  |  |
| SIS | Psychometric properties not evaluated | 17 | UL-MAS | Psychometric properties not evaluated | 7 |
|  | Conference proceedings | 9 |  | Conference proceedings | 0 |
|  | Not conducted in English | 4 |  | Not conducted in English | 4 |
|  | Modified or incorrect measure | 25 |  | Modified or incorrect measure | 1 |
|  | Not original research | 1 |  | Not original research | 0 |
|  | Protocol only | 0 |  | Protocol only | 0 |
|  | Participants outside criteria | 0 |  | Participants outside criteria | 0 |

ARAT = Action Research Arm Test, ArmA = Arm Activity Measure, AQoL = Assessment of Quality of Life, BI = Barthel Index, CMSA = Chedoke-McMaster Stroke Assessment, DAS = Disability Assessment Scale, EQ-5D = EuroQol – 5 dimension, FAT = Frenchay Arm Test, mFAT = modified Frenchay Arm Test, FIM = Functional Independence Measure, GAS = Goal Attainment Scale, GAS – 10pt = Goal Attainment Scale – 10 point, Global Ax = Global Assessment Scale, KleinBell ADL = Klein-Bell Activities of Daily Living scale, LASIS = Leeds Adult Spasticity Impact Scale, SF-36 = Medical Outcome Study 36-Item Short-Form Health Survey, MAL = Motor Activity Log, MAL-5 = Motor Activity Log - 5, MAL-28 = Motor Activity Log - 28, MI = Motricity Index, NHPT = Nine Hole Peg Test, OHS = Oxford Handicap Scale, PDS/CBS = Patient Disability Scale / Carer Burden Scale, RMA = Rivermead Motor Assessment, RMA-UL =Rivermead Motor Assessment - Upper Limb, SA-SIP =Stroke-Adapted Version of the Sickness Impact Profile, SIS = Stroke Impact Scale, UL MAS = Upper Limb Motor Assessment Scale.
