# Supplementary material for: Psychometric properties of measures of upper limb activity performance in adults with and without spasticity undergoing neurorehabilitation–A systematic review
Source: PLoS One. 2021 Feb 11;16(2):e0246288. doi: 10.1371/journal.pone.0246288 (PMC7877653; doi:10.1371/journal.pone.0246288)
Supplement: S2 Table — This file lists all included studies and methodological quality and quality criteria ratings. (DOCX) [file pone.0246288.s004.docx]

| **S2 Table.** Methodological quality and quality criteria ratings | | | | | | | | | | | |
| --- | --- | --- | --- | --- | --- | --- | --- | --- | --- | --- | --- |
| **Action Research Arm Test** | **Content validity** | **Structural validity** | **Internal consistency** | **Reliability** | | | | | **Measurement error** | **Construct validity** | **Responsiveness** |
|  |  |  |  | **Inter** | | **Intra** | | **Retest** |  |  |  |
| *Beebe & Lang (2009) [39]* |  |  |  |  | |  | |  |  | *Very good*  *- (3/5)* | *Doubtful*  *+ (2/2)* |
| *Burridge et al., (2009) [46]* |  |  |  |  | |  | |  |  | *Adequate*  *- (4/8)* |  |
| De Weerdt & Harrison (1985) [58] |  |  |  |  | |  | |  |  | Very good  + (1/1) | Inadequate  + (1/1) |
| Dromerick et al., (2006) [64] |  |  |  |  | |  | |  |  | Very good  - (2/3) |  |
| *Fleming et al., (2014) [76]* |  |  |  |  | |  | |  |  | *Adequate*  *+ (1/1)* |  |
| Lang et al., (2008) [114]* |  |  |  |  | |  | |  |  |  |  |
| *Lang et al., (2006) [115]* |  |  |  |  | |  | |  |  | *Very good*  *- (5/7)* | *Doubtful*  *+ (2/2)* |
| Lyle (1981) [121] | Inadequate | Inadequate  + |  |  | |  | |  |  |  |  |
| Morris et al., (2013) [130] |  |  |  |  | |  | |  |  | Doubtful  - (1/2) |  |
| Rabadi & Rabadi (2006) [141] |  |  |  |  | |  | |  |  | Very good  – (1/2) | Adequate  + (1/1) |
| Rand & Eng (2015) [143] |  |  |  |  | |  | |  |  | Very good  + (1/1) |  |
| Yozbatiran et al., (2008) [173] |  |  |  | Adequate  + | | Adequate  + | |  |  |  |  |
| **Arm Activity Measure** | **Content validity** | **Structural validity** | **Internal consistency** | **Reliability** | | | | | **Measurement error** | **Construct validity** | **Responsiveness** |
|  |  |  |  | **Inter** | | **Intra** | | **Retest** |  |  |  |
| *Ashford et al., (2015) [31]* | *Very good*  *+/-* |  |  |  | |  | |  |  |  |  |
| *Ashford et al., (2016) [32]* |  | *Adequate*  *+* |  |  | |  | |  |  |  |  |
| *Ashford et al., (2014) [33]* |  |  |  |  | |  | |  |  |  | *Inadequate*  *+ (2/2)* |
| *Ashford et al., (2013) [177]* | *Adequate*  *+* |  |  |  | |  | |  |  |  |  |
| *Ashford et al., (2013) [35]* |  | *Adequate*  *+* | *Very good*  *+* |  | |  | | *Adequate*  *+* |  | *Inadequate*  *+ (2/2)* | *Adequate*  *+ (2/2)* |
| **Assessment of Quality of Life** | **Content validity** | **Structural validity** | **Internal consistency** | **Reliability** | | | | | **Measurement error** | **Construct validity** | **Responsiveness** |
|  |  |  |  | **Inter** | | **Intra** | | **Retest** |  |  |  |
| Hawthorne et al., (2009) [90] |  |  |  |  | |  | |  |  | Very good  + (1/1) |  |
| Hawthorne et al., (1999) [91] | Inadequate |  |  |  | |  | |  |  |  |  |
| Sturm et al., (2002) [156]** |  |  |  |  | |  | |  |  | Adequate  + (1/1) |  |
| **Barthel Index** | **Content validity** | **Structural validity** | **Internal consistency** | **Reliability** | | | | | **Measurement error** | **Construct validity** | **Responsiveness** |
|  |  |  |  | **Inter** | | **Intra** | | **Retest** |  |  |  |
| Ali et al., (2013) [29] |  |  |  |  | |  | |  |  | Adequate  – (0/1) |  |
| Filiatrault et al., (1991) [73] |  |  |  |  | |  | |  |  | Adequate  + (2/2) | Inadequate  ? |
| Kwon et al., (2004) [112] |  |  |  |  | |  | |  |  | Very good  + (2/2) |  |
| Mahoney & Barthel (1965) [124] | Inadequate |  |  |  | |  | |  |  |  |  |
| Wallace et al., (2002) [165] |  |  |  |  | |  | |  |  |  | Doubtful  - (0/1) |
| Wellwood et al., (1995) [167] |  |  |  |  | |  | |  |  | Doubtful  + (1/1) |  |
| **Barthel Index (Collin & Wade)** | **Content validity** | **Structural validity** | **Internal consistency** | **Reliability** | | | | | **Measurement error** | **Construct validity** | **Responsiveness** |
|  |  |  |  | **Inter** | | **Intra** | | **Retest** |  |  |  |
| Barer & Murphy (1993) [36] |  | Inadequate  ? |  |  | |  | |  |  | Inadequate  + (2/2) |  |
| Collin et al., (1988) [50] | Inadequate |  |  | Inadequate  ? | |  | |  |  |  |  |
| Dennis et al., (2000) [57] |  |  |  |  | |  | |  |  | Inadequate  + (4/4) |  |
| Green et al., (2001) [84] |  |  |  |  | |  | | Inadequate  ? | Adequate  + |  |  |
| Houlden et al., (2006) [98] |  |  |  |  | |  | |  |  |  | Doubtful  + (1/1) |
| Sarker et al., (2012) [150] |  |  |  |  | |  | |  |  | Doubtful  + (2/2) |  |
| van der Putten et al., (1999) [160] |  |  |  |  | |  | |  |  |  | Doubtful  – (1/2) |
| Wade & Hewer (1987) [164] |  | Adequate  + |  |  | |  | |  |  | Doubtful  + (1/1) |  |
| Wilkinson et al., (1997) [168] |  |  |  |  | |  | |  |  | Doubtful  + (8/8) |  |
| **Chedoke-McMaster Stroke Assessment** | **Content validity** | **Structural validity** | **Internal consistency** | **Reliability** | | | | | **Measurement error** | **Construct validity** | **Responsiveness** |
|  |  |  |  | **Inter** | | **Intra** | | **Retest** |  |  |  |
| Dang et al., (2011) [55] |  |  |  |  | |  | |  |  | Doubtful  - (1/2) |  |
| Gowland (1990) [81] | Inadequate |  |  |  | |  | |  |  |  |  |
| Gowland et al., (1993) [82] |  |  |  | II very good +  AI adequate + | | Very good  + | | Adequate  - |  | Adequate  + (4/4) | Adequate  + (1/1) |
| Moreland et al., (1993) [129] | Inadequate |  |  |  | |  | |  |  |  |  |
| **Disability Assessment Scale** | **Content validity** | **Structural validity** | **Internal consistency** | **Reliability** | | | | | **Measurement error** | **Construct validity** | **Responsiveness** |
|  |  |  |  | **Inter** | | **Intra** | | **Retest** |  |  |  |
| *Brashear et al., (2002) [43]* | *Inadequate*  *?* |  |  | *Adequate*  *-* | | *Adequate*  *?* | |  |  |  |  |
| *Doan et al., (2012) [59]* |  |  |  |  | |  | |  |  | *Adequate*  *+ (2/2)* |  |
| **EuroQol – 5 Dimension** | **Content validity** | **Structural validity** | **Internal consistency** | **Reliability** | | | | | **Measurement error** | **Construct validity** | **Responsiveness** |
|  |  |  |  | **Inter** | | **Intra** | | **Retest** |  |  |  |
| Alderman et al., (2001) [28] |  |  |  |  | |  | |  |  | Adequate  - (0/4) |  |
| Barton et al., (2008) (a) [38] |  |  |  |  | |  | |  |  | Very good  + (1/1) |  |
| Barton et al., (2008) (b) [37] |  |  |  |  | |  | |  |  | Adequate  + (1/1) |  |
| *Doan et al., (2012) [59]* |  |  |  |  | |  | |  |  | *Adequate*  *+ (2/2)* |  |
| Dorman et al., (1999) [61] |  |  |  |  | |  | |  |  | Adequate  + (1/1) |  |
| Dorman et al., (1998) [62] |  |  |  |  | |  | | Doubtful  + patient  - proxy |  |  |  |
| Dorman et al., (1997) [63] |  |  |  |  | |  | |  |  | Doubtful  + (6/7) |  |
| Fisk et al., (2005) [74] |  |  |  |  | |  | | Adequate  + |  | Adequate  + (6/6) |  |
| *Gillard et al., (2015) [79]* |  |  |  |  | |  | |  |  | *Adequate*  *+ (1/1)* |  |
| Kohn et al., (2014) [107] |  |  |  |  | |  | |  |  | Adequate  - (1/2) |  |
| Kuspinar & Mayo (2013) [109] | Doubtful  +/- |  |  |  | |  | |  |  | Adequate  + (2/2) |  |
| Kuspinar et al., (2014) [108] |  |  |  |  | |  | |  |  | Very good  + (1/1) |  |
| Moore et al., (2004) [128] |  |  |  |  | |  | |  |  | Inadequate  - (1/3) |  |
| Nicholl et al., (2001) [132] |  |  |  |  | |  | |  |  | Doubtful  - (0/2) |  |
| Peters et al., (2014) [136] |  |  |  |  | |  | |  |  |  | Inadequate  – (0/2) |
| Pickard et al., (2005) [137] |  |  |  |  | |  | |  |  |  | Adequate  + (11/13) |
| Salter et al., (2008) [149] | Doubtful  ? |  |  |  | |  | |  |  |  |  |
| Williams (1990) [170] | Inadequate |  |  |  | |  | |  |  |  |  |
| Xie et al., (2006) [172] |  |  |  |  | |  | |  |  | Very good  + (1/1) |  |
| **modified Frenchay Arm Test** | **Content validity** | **Structural validity** | **Internal consistency** | **Reliability** | | | | | **Measurement error** | **Construct validity** | **Responsiveness** |
|  |  |  |  | **Inter** | | **Intra** | | **Retest** |  |  |  |
| (a) Heller et al., (1987) [95] |  |  |  | Doubtful  ? | |  | | Doubtful  ? |  |  |  |
| ^(b)^ Heller et al., (1987) [95] |  |  |  |  | |  | |  |  | Doubtful  - (0/1) |  |
| **Functional Independence Measure** | **Content validity** | **Structural validity** | **Internal consistency** | **Reliability** | | | | | **Measurement error** | **Construct validity** | **Responsiveness** |
|  |  |  |  | **Inter** | **Intra** | | **Retest** | |  |  |  |
| Brown et al., (2015) [45] |  |  |  |  | |  | |  |  | Very good  + (3/3) |  |
| Corrigan et al., (2014) [51] |  |  |  |  | |  | |  |  | Doubtful +  (9/9) |  |
| Cullen et al., (2014) [53] |  |  |  |  | |  | |  |  | Very good  + (2/2) |  |
| Cuthbert et al., (2015) [54] |  |  |  |  | |  | |  |  | Adequate  – (0/1) |  |
| Egan et al., (2014) [71] |  |  |  |  | |  | |  |  | Doubtful  + (1/1) |  |
| Grant et al., (2014) [83] |  |  |  |  | |  | |  |  | Adequate  – (0/1) |  |
| Hall et al., (1993) [87] |  |  |  |  | |  | |  |  | Inadequate  + (6/6) |  |
| Hamilton & Granger (1994) [88] |  |  |  | Doubtful  + | |  | |  |  |  |  |
| Heinemann et al., (1993) [93] |  | Adequate  + |  |  | |  | |  |  |  |  |
| Heinemann et al., (1994) [94] |  | Adequate  + |  |  | |  | |  |  |  |  |
| Heinemann et al., (1997) [92] |  |  |  |  | |  | |  |  | Inadequate  + (1/1) |  |
| Houlden et al., (2006) [98] |  |  |  |  | |  | |  |  |  | Doubtful  - (2/3) |
| Keith et al., (1987) [106] | Inadequate |  |  |  | |  | |  |  |  |  |
| Kuys et al., (2009) [110] |  |  |  |  | |  | |  |  | Adequate  - (0/1) |  |
| Oczkowski et al., (1993) [133] |  |  |  |  | |  | |  |  | Adequate  + (1/1) |  |
| Ouellette et al., (2015) [135] |  |  |  |  | |  | |  |  | Doubtful  – (0/1) |  |
| *Rabadi & Vincent (2013) [142]* |  |  |  |  | |  | |  |  | *Adequate*  *+ (1/1)* | *Doubtful*  *+ (1/1)* |
| Sharrack et al., (1999) [152] |  | Inadequate  + | Adequate  + | Adequate  + | | Adequate  + | |  |  | Adequate  - (0/1) | Doubtful  + (1/1) |
| Stineman et al., (1996) [154] |  | Adequate  + | Adequate  + |  | |  | |  |  |  |  |
| van der Putten et al., (1999) [160] |  |  |  |  | |  | |  |  |  | Doubtful  - (1/2) |
| **Goal Attainment Scale** | **Content validity** | **Structural validity** | **Internal consistency** | **Reliability** | | | | | **Measurement error** | **Construct validity** | **Responsiveness** |
|  |  |  |  | **Inter** | | **Intra** | | **Retest** |  |  |  |
| Bovend’Eerdt et al., (2011) [42] |  |  |  | Adequate  - | |  | |  | Adequate  ? |  |  |
| Brock et al., (2009) [44] |  |  |  |  | |  | |  |  | Inadequate  + (4/5) |  |
| Doig et al., (2010) [60] |  |  |  |  | |  | |  |  | Adequate  + (1/1) | Inadequate  + (1/1) |
| Joyce et al., (1994) [103] |  |  |  | Doubtful  ? | |  | |  |  | Doubtful  - (4/7) |  |
| Khan et al., (2008) [105] |  |  |  |  | |  | |  |  | Very good  - (1/3) | Doubtful  + (2/2) |
| Lannin (2003) [116] |  |  |  |  | |  | |  |  |  | Doubtful  + (1/1) |
| Malec (1999) [125] |  |  |  |  | |  | |  |  | Very good  + (1/1) |  |
| Malec et al., (1991) [126] |  |  |  |  | |  | |  |  | Very good  + (4/4) |  |
| *Turner-Stokes et al., (2010) [157]* |  |  |  |  | |  | |  |  | *Doubtful*  *- (3/7)* |  |
| **Motor Activity Log** | **Content validity** | **Structural validity** | **Internal consistency** | **Reliability** | | | | | **Measurement error** | **Construct validity** | **Responsiveness** |
|  |  |  |  | **Inter** | | **Intra** | | **Retest** |  |  |  |
| Chen et al., (2012) [48] |  |  |  |  | |  | |  | Adequate  ? |  |  |
| Dromerick et al., (2006) [64] |  |  |  |  | |  | |  |  | Very good  - (1/2) |  |
| *Harris & Eng (2007) [89]* |  |  |  |  | |  | |  |  | *Very good*  *- (3/7)* |  |
| Uswatte et al., (2006) [159] |  | Inadequate  ? |  |  | |  | |  |  |  |  |
| Uswatte & Taub (2005) [158] | Inadequate |  |  |  | |  | |  |  |  |  |
| **Motor activity Log-28** | **Content validity** | **Structural validity** | **Internal consistency** | **Reliability** | | | | | **Measurement error** | **Construct validity** | **Responsiveness** |
|  |  |  |  | **Inter** | | **Intra** | | **Retest** |  |  |  |
| Uswatte et al., (2006) [159] | Inadequate  ? | Inadequate  ? | Very good + |  | |  | | Adequate  Patient +  Proxy - |  | Inadequate  Patient + (3/4)  Proxy – (2/4) |  |
| **Motricity Index** | **Content validity** | **Structural validity** | **Internal consistency** | **Reliability** | | | | | **Measurement error** | **Construct validity** | **Responsiveness** |
|  |  |  |  | **Inter** | | **Intra** | | **Retest** |  |  |  |
| Bohannon (1999) [41] |  |  | Doubtful  + |  | |  | |  |  | Inadequate  - (0/1) |  |
| Collin & Wade (1990) [49] |  |  |  | Doubtful  ? | |  | |  |  | Adequate  + (1/1) |  |
| Demeurisse et al., (1980) [56]^ | Inadequate |  |  |  | |  | |  |  |  |  |
| Jacob-Lloyd et al., (2005) [99] |  |  |  |  | |  | |  |  | Very good  – (1/2) | Doubtful  - (0/1) |
| Stone et al., (1993) [155] |  |  |  |  | |  | |  |  | Doubtful  + (1/1) |  |
| Wade & Hewer (1987) [164] |  |  |  |  | |  | |  |  | Doubtful  + (1/1) |  |
| **Nine Hole Peg Test** | **Content validity** | **Structural validity** | **Internal consistency** | **Reliability** | | | | | **Measurement error** | **Construct validity** | **Responsiveness** |
|  |  |  |  | **Inter** | | **Intra** | | **Retest** |  |  |  |
| *Beebe & Lang (2009) [39]* |  |  |  |  | |  | |  |  | *Very good*  *– (3/5)* | *Doubtful*  *+ (2/2)* |
| Benedict et al., (2011) [40] |  |  |  |  | |  | |  |  | Adequate  + (6/8) |  |
| Costelloe et al., (2008) [52] |  |  |  |  | |  | |  |  | Adequate  - (2/3) |  |
| Goodkin et al., (1988) [80] |  |  |  |  | |  | |  |  | Adequate  + (2/2) |  |
| ^(a)^ Heller et al., (1987) [95] |  |  |  | Doubtful  ? | |  | | Doubtful  ? |  |  |  |
| ^(b)^ Heller et al., (1987) [95] |  |  |  |  | |  | |  |  | Doubtful  + (1/1) |  |
| Jacob-Lloyd et al., (2005) [99] |  |  |  |  | |  | |  |  | Very good  - (1/2) | Doubtful  + (1/1) |
| Morris et al., (2013) [130] |  |  |  |  | |  | |  |  | Doubtful  - (0/1) |  |
| Poole et al., (2010) [140] |  |  |  |  | |  | |  |  | Adequate  - (6/10) |  |
| Schwid et al., (2002) [151] |  |  |  |  | |  | |  | Adequate  + |  |  |
| **Oxford Handicap Scale** | **Content validity** | **Structural validity** | **Internal consistency** | **Reliability** | | | | | **Measurement error** | **Construct validity** | **Responsiveness** |
|  |  |  |  | **Inter** | | **Intra** | | **Retest** |  |  |  |
| Rigby et al., (2009) [145] |  |  |  |  | |  | |  |  | Doubtful  - (0/1) |  |
| Simon et al., (2008) [153] |  |  |  |  | |  | |  |  | Adequate +  (2/2) |  |
| **Rivermead Motor Assessment** | **Content validity** | **Structural validity** | **Internal consistency** | **Reliability** | | | | | **Measurement error** | **Construct validity** | **Responsiveness** |
|  |  |  |  | **Inter** | | **Intra** | | **Retest** |  |  |  |
| Adams et al., (1997) [27] |  | Inadequate  - |  |  | |  | |  |  |  |  |
| Adams et al., (1997) [26] |  | Inadequate  - |  |  | |  | |  |  |  |  |
| Jones (1998) [102] |  |  |  |  | |  | |  |  | Very good  +(3/3) |  |
| Lincoln & Leadbitter (1979) [118] | Inadequate |  |  |  | |  | |  |  |  |  |
| Sackley (1990) [148] |  |  |  |  | |  | |  |  | Adequate  + (2/2) |  |
| **Rivermead Motor Assessment – Upper Limb** | **Content validity** | **Structural validity** | **Internal consistency** | **Reliability** | | | | | **Measurement error** | **Construct validity** | **Responsiveness** |
|  |  |  |  | **Inter** | | **Intra** | | **Retest** |  |  |  |
| Adams et al., (1997) [27] |  | Inadequate  + |  |  | |  | |  |  |  |  |
| Adams et al., (1997) [26, 27] |  | Inadequate  - |  |  | |  | |  |  |  |  |
| Collin et al., (1990) [49] |  |  |  |  | |  | |  |  | Adequate  + (1/1) |  |
| Lincoln & Leadbitter (1979) [118] | Inadequate |  |  |  | |  | |  |  |  |  |
| Morris et al., (2013) [130] |  |  |  |  | |  | |  |  | Doubtful  - (0/1) |  |
| Sackley (1990) [148] |  |  |  |  | |  | |  |  | Adequate  + (2/2) |  |
| **SA-SIP30** | **Content validity** | **Structural validity** | **Internal consistency** | **Reliability** | | | | | **Measurement error** | **Construct validity** | **Responsiveness** |
|  |  |  |  | **Inter** | | **Intra** | | **Retest** |  |  |  |
| *Doan et al., (2012) [59]* |  |  |  |  | |  | |  |  | *Adequate*  *+ (1/1)* |  |
| Edwards et al., (2006) [70] |  |  |  |  | |  | |  |  | Adequate  + (2/2) |  |
| Salter et al., (2008) [149] | Doubtful  ? |  |  |  | |  | |  |  |  |  |
| van Straten et al., (1997) [161]^ | Inadequate |  |  |  | |  | |  |  |  |  |
| **SF-36** | **Content validity** | **Structural validity** | **Internal consistency** | **Reliability** | | | | | **Measurement error** | **Construct validity** | **Responsiveness** |
|  |  |  |  | **Inter** | | **Intra** | | **Retest** |  |  |  |
| Anderson et al., (1996) [30] |  |  | Doubtful  + |  | |  | |  |  | Adequate  - (1/2) |  |
| Dorman et al., (1999) [61] |  |  |  |  | |  | |  |  | Adequate  + (1/1) |  |
| Dorman et al., (1998) [62] |  |  | Very good  + |  | |  | | Patient adequate +  Proxy  Adequate  - |  |  |  |
| Duncan et al., (1997) [68] |  |  |  |  | |  | |  |  | Doubtful  + (1/1) |  |
| Findler et al., (2001) [75] |  |  |  |  | |  | |  |  | Adequate  + (2/2) |  |
| Freeman et al., (2000) [77] |  |  | Very good  + |  | |  | |  |  | Adequate  + (7/7) | Doubtful  - (0/1) |
| Freeman et al., (1996) [78] |  |  |  |  | |  | |  |  | Very good  - (2/3) |  |
| Guilfoyle et al., (2010) [85] |  | Very good  - | Very good  + |  | |  | |  |  | Doubtful  + (1/1) |  |
| Hagen et al., (2003) [86] |  |  | Very good  + |  | |  | |  |  | Inadequate  - (1/3) | Doubtful  - (0/2) |
| Hermann et al., (1996) [96] |  |  |  |  | |  | |  |  | Very good  - (1/2) |  |
| Hobart et al., (2002) [97] |  | Adequate  ? | Very good  + |  | |  | |  |  |  |  |
| Mackenzie et al., (2002) [122] |  | Very good  + |  |  | |  | |  |  | Adequate  - (0/1) |  |
| Madden et al., (2006) [123] |  |  |  |  | |  | |  |  | Very good  - (0/1) | Inadequate  - (0/1) |
| Moore et al., (2004) [128] |  |  |  |  | |  | |  |  | Inadequate  - (1/6) |  |
| Murrell et al., (1999) [131] |  |  |  |  | |  | | Doubtful  ? |  |  |  |
| O’Mahony et al., (1998) [134]** |  |  |  |  | |  | |  |  |  |  |
| Pittock et al., (2004) [139] |  |  |  |  | |  | |  |  | Very good  - (1/2) |  |
| Riazi et al., (2003) [144] |  |  |  |  | |  | |  |  | Very good  + (1/1) |  |
| Robinson et al., (2009) [146] |  |  |  |  | |  | |  |  | Adequate  - (0/1) |  |
| Salter et al., (2008) [149] | Doubtful  ? |  |  |  | |  | |  |  |  |  |
| Vickrey et al., (1995) [163] |  |  |  |  | |  | |  |  | Adequate  + (1/1) |  |
| Vickrey et al., (1997) [162] |  |  | Very good  + |  | |  | | Adequate  + |  | Very good  - (4/8) |  |
| Ware & Sherbourne (1992) [166] | Inadequate |  |  |  | |  | |  |  |  |  |
| Williams et al., (1999) [169] |  |  |  |  | |  | |  |  | Inadequate  - (0/1) |  |
| **Stroke Impact Scale** | **Content validity** | **Structural validity** | **Internal consistency** | **Reliability** | | | | | **Measurement error** | **Construct validity** | **Responsiveness** |
|  |  |  |  | **Inter** | | **Intra** | | **Retest** |  |  |  |
| Duncan et al., (2003) [65] | Inadequate | Very good  + |  |  | |  | |  |  |  |  |
| Duncan et al., (2002) [66] |  |  |  | Adequate  ? | |  | |  |  | Adequate  + (8/8) |  |
| Duncan et al., (2005) [67] |  |  | Very good  + |  | |  | | Doubtful  + |  |  |  |
| Duncan et al., (1999) [69]^^ | Inadequate |  |  |  | |  | |  |  |  |  |
| Eriksson et al., (2013) [72] |  |  |  |  | |  | |  |  | Very good  + (4/5) |  |
| Jenkinson et al., (2013) [100] |  | Very good  + | Very good  + |  | |  | |  |  |  |  |
| Kwon et al., (2006) [111] |  |  |  |  | |  | |  |  | Adequate  + (4/4) |  |
| Lai et al., (2002) [113] |  |  |  |  | |  | |  |  | Doubtful  + (1/1) |  |
| Salter et al., (2008) [149] | Doubtful  ? |  |  |  | |  | |  |  |  |  |
| Wolf & Koster (2013) [171] |  |  |  |  | |  | |  |  | Doubtful  + (1/1) |  |
| **Upper Limb – Motor Assessment Scale** | **Content validity** | **Structural validity** | **Internal consistency** | **Reliability** | | | | | **Measurement error** | **Construct validity** | **Responsiveness** |
|  |  |  |  | **Inter** | | **Intra** | | **Retest** |  |  |  |
| Carr et al., (1985) [47] | Inadequate |  |  | Inadequate  ? | |  | |  |  |  |  |
| Johnson & Selfe (2004) [101] |  |  | Very good  + |  | |  | |  |  |  |  |
| Khan et al., (2013) [104] |  | Adequate  + |  |  | |  | |  |  | Very good  + (1/1) |  |
| Kuys et al., (2009) [110] |  |  |  |  | |  | |  |  | Adequate  - (0/1) |  |
| Lannin (2004) [117] |  | Very good  ? | Very good  + |  | |  | |  |  |  |  |
| Loewen & Anderson (1988) [119] |  |  |  | Doubtful  ? | | Doubtful  ? | |  |  |  |  |
| Loewen & Anderson (1990) [120] |  |  |  |  | |  | |  |  | Adequate  - (2/6) |  |
| Miller et al., (2010) [127] |  | Very good  + | Very good  + |  | |  | |  |  |  |  |
| Pickering et al., (2010) [138] |  | Inadequate  - |  |  | |  | |  |  |  |  |
| Sabari et al., (2005) [147] |  | Doubtful  - |  |  | |  | |  |  |  |  |
