# Supplementary material for: Psychometric properties of measures of upper limb activity performance in adults with and without spasticity undergoing neurorehabilitation–A systematic review
Source: PLoS One. 2021 Feb 11;16(2):e0246288. doi: 10.1371/journal.pone.0246288 (PMC7877653; doi:10.1371/journal.pone.0246288)
Supplement: S3 Table — This file provides a summary of results for all included studies. (DOCX) [file pone.0246288.s005.docx]

| **S3 Table.** Summary of study results | | | | |  |  |  |  |  |  |  |  |
| --- | --- | --- | --- | --- | --- | --- | --- | --- | --- | --- | --- | --- |
| Measurement tool | Content validity | Structural validity | Internal consistency | Reliability | | | | Measurement error | Construct validity | Responsiveness | Interpretability | References |
|  |  |  |  | Inter | | Intra | Retest |  |  |  |  |  |
| ARAT  (Obs perf measure of upper limb activity) | Modification of the UEFT | Guttman Scaling criteria met | - | ICC 0.99 | | ICC 0.99 | - | - | Measures of;   - Activity and UL dexterity r = -0.25-0.95 - Global measures of function r = 0.2 – 0.6 - Impairment r = 0.03–0.86   Predicting UL use R^2^ = 0.6 (P<0.001)–0.776, (P<0.01)  Not predictive HRQoL | ES = 0.52–1.018  RR= 5.20–7.067  SRM = 0.68  R^2^ = .56, P<.001  T=1, n=28, z=4.60 | MCID 12, 17  Floor effect 0%  Ceiling effect 0-41% | [39, 46, 58, 64, 76, 115, 121, 130, 141, 143, 173] |
|  |  |  |  |  | |  |  |  |  |  |  |  |
| ArmA  (Self-report measure of upper limb activity – active and passive) | Tool development methodology supports content validity.  Items derived from patient goals (categorised into passive function, active function, symptoms, cosmesis, impairment) informed standardised measure development. | Unidimensional passive and active scales.  Passive subscale conforms to Rasch model.  Able to differentiate at least two groups of patients. | Passive subscale α = 0.85, active subscale α = 0.96 |  | |  | Weighted kappa passive subscale 0.90 (CI 0.68 – 1.12), active subscale 0.93 (CI 0.71 – 1.15) |  | Passive subscale with;   - passive items on LASIS Rho 0.50; p = 0.01, - active items LASIS Rho 0.02; p = 0.9   Active function subscale with;   - active LASIS Rho 0.48; p = 0.01 - DASH active items Rho 0.63 p = 0.01 - passive LASIS items Rho 0.23 p = 0.078 | Significant difference between responder and non responder groups for passive function subscale (8 wks) U = 98.5; p = 0.01), not shown for the active function subscale (U=163.4; p=0.35) as expected.  More responsive than LASIS, DASH active items, Barthel Index .  Detected change post focal spasticity intervention | Ceiling effect (37%) active function subscale.  No floor effect on either subscale. | [8, 31-33, 177] |
|  |  |  |  |  | |  |  |  |  |  |  |  |
| AQoL  (Self-report measure of global HRQoL) | - | - | - | - | | - | - | - | Measures of;   - Handicap LHS r = 0.83 - Disability BI r = 0.77 - Impairment r = -0.69 - Mood r = -0.63- -0.60 - Global measure of function SF-36 r = 0.15–0.83   Discriminates between stroke types more efficiently than SF-36  Sensitive in TBI ES -0.80  Predictor of death and/or institutionalization | - | No floor or ceiling effect (1-2%) | [90, 91, 156] |
|  |  |  |  |  | |  |  |  |  |  |  |  |
| BI  (Self-report or obs perf measure of global ADL) | - | - | - | - | | - | - | - | Greater associated with proxy ratings of QoL than patient (r = 0.68)   - m-FIM r_s_=0.9479 *p*<0.0001 - MRS r_s_=-0.8856 *p*<0.0001. - Fugl-Meyer r rho = 0.60 - Functional test for the hemiparetic UL Rho = .61 - disability r=-0.73 *p*<0.001. | Kendall’s statistic W = 0.39  t =0.63 | Ceiling effect | [29, 73, 112, 165, 167] |
|  |  |  |  |  | |  |  |  |  |  |  |  |
| BI (C&W)  (Self-report or obs perf measure of global ADL) | - | One major factor.  Guttman scaling criteria not met. | - | Disagreement of 1 - 9 points between self-report, nurse, 2 skilled observers.  Agreement lower for transfers, feeding, dressing, grooming, toileting. | | - | >75% agreement, kappa -0.99 to 0.81. | 0.4 (95%CI 0.01-0.90)  Difference of 4/20 points likely to represent genuine change | Predicts DC destination and LOS ≥ 0.70,  Stronger relationships with measures of disability and physical abilities  Measures of;   - Activity = 0.73-0.83 - Disability r_s_ = 0.73-0.8 all P<0.01. - Global measures of function r_s_ = 0.33 -0.81, - Mental health r_s_ = -0.2 - -0.4 | TBI  FIM change scores r^2^ = 0.733  MS  ES = 0.37  Stroke  ES = 0.52-0.95 | Floor and ceiling effects | [36, 50, 57, 84, 98, 150, 160, 164, 168] |
|  |  |  |  |  | |  |  |  |  |  |  |  |
| CMSA  (Obs perf measure of impairment and mobility) | - | - | - | ICC = 0.88 (95% CI 0.76–0.94) to 0.99 (95% CI 0.98–1.00) | | ICC 0.93 (95% CI 0.85–0.96) to 0.98 (95% CI 0.95–0.99) | Disability Total 0.98 (95% CI 0.95–0.99) | - | - Fugl-Meyer Test r = 0.95 p<0.001 - FIM r = 0.79, p<0.05 (disability inventory) - Gowlands predictive equations; not supported | CMSA disability inventory (0.53 F 37.25 p<0.001) more responsive than FIM (0.39 F 19.40 p<0.001). | - | [55, 82] |
|  |  |  |  |  | |  |  |  |  |  |  |  |
| DAS  (Self-report or obs perf measure of upper limb activity – active and passive) | - | - | - | Kendall W Hygiene 0.626 (95% CI 0.297–1.00 p<0.001), dressing 0.494 (95% CI 0.234–1.00 p<0.001) limb position 0.557 (95% CI 0.264–1.00 p<0.001) pain 0.772 (95% CI 0.366 – 1.00 p<0.001) | | Weighted Kappa hygiene 0.520 (95% CI 0.239 – 0.802), P .994, Dressing 0.530 (95% CI 0.278 – 0.782) P 0.478, Limb position 0.775 (95% CI 0.560-0.991) P 0.998, Pain 0.776 (95% CI 0.533–1.0) P 0.992. | - | - | Increasing disability in DAS was associated with diminishing EQ-5D index scores (P < .002) Increasing disability is associated with reduction in HRQoL and caregiver burden (P<.05) | - | - | [43, 59] |
|  |  |  |  |  | |  |  |  |  |  |  |  |
| EQ-5D  (Self-report measure of global HRQoL) | Includes   - 4/10 domains most important to people with MS and 2/7 domains not prioritized by people with MS - 6/9 items related to domains considered important for people post-stroke. | - | - | - | | - | ICC = 0.63–0.80 (individual domains)  ICC = 0.81-0.86 (overall)  Self-report more reliable than proxy | - | No significant differences between staff and participant ratings  Measures of   - global function r = 0.33 - 0.77 - UL activity/dexterity r = 0.56 - disability (P<.002) - self-care r = 0.64 - activities r = 0.60 - carer burden (P<.05) - cognition/psychological r = 0-0.56 - Mobility r = -0.69-61 - pain r = 0.71 - general health status r = 0.80 - Less sensitive than FAMS and SF-54   EQ5D index stronger association with measures of disability and ADL  VAS stronger correlations with changes in mental functioning  Discriminant validity;   - baseline stroke severity and type - spasticity. Mean difference 0.07 (CI -0.12 to -0.03) - stroke (average 6.9% lower index, 7.2% lower VAS) - functional walking capacity and general health perception | EQ-5D mean change -0.01 (95%CI -0.03 to 0.01),  VAS mean change -1.88 (95%CI -5.12 to 1.37)  No significant change over 12 mo.  EQ5D greatest change scores amongst generic measures. | Index floor 0%, ceiling 13.3%  VAS  floor 0% % ceiling 3%.  Individual subscales: floor effect 2-34%, ceiling effect 7-68% | [28, 37, 38, 59, 61-63, 74, 79, 107-109, 128, 132, 136, 137, 149, 172] |
|  |  |  |  |  | |  |  |  |  |  |  |  |
| FAT  (Obs perf measure of upper limb activity) | - | - | - | - | | - | - | - | - | - | - | - |
|  |  |  |  |  | |  |  |  |  |  |  |  |
| mFAT  (Obs perf measure of upper limb activity) | - |  | - | Rho = 0.83–0.99 | | Rho = 0.68–0.90 | Rho = 0.75–0.99 | - | Less sensitive than NHPT 52% within normal NHPT scores. | - | Floor effect 30%  Ceiling effect 34% | [95] |
|  |  |  |  |  | |  |  |  |  |  |  |  |
| FIM  (Obs perf measure of global ADL) | - | Two factor (motor and cognitive) structure supported – (89.4% of total variance explained).  3 or 4 factor structure proposed (.>50% of variance explained) | FIM total α = 0.94 – 0.98, FIM motor α = 0.93 – 0.97, FIM cognitive α = 0.93 – 0.94 | FIM total ICC = 0.99, FIM motor ICC = 0.96 FIM cognitive ICC = 0.91 | | FIM total ICC = 0.94 | - | - | Predictive of;   - LOS - Discharge destination (X_2_ = 69.4, P<0.001, AUC = 0.76, sensitivity = 0.76, specificity = 0.64)., - Minutes and type of assistance and supervision(p<0.0001), (p<0.0032), (p<0.0063) - Predicting RTD sensitivity 72%, specificity 73%, ROC 0.71 - More superior prediction than SF-36   Measures of   - Activity r = 0.21 P<.01 to 0.54 P<.001 - Disability r = 0.64-0.96 - Work r=-0.59, p<0.001–0.64 p=0.001 - Independence r=-0.44 p=0.001 - Global measures of function r = 0.63–0.92 - Robotic measures = -0.21- -0.79 - QoL r = 0.41-0.86 - Depression r = 0.27- -.43 (p = .001)   Low correlations with;   - DC walking speed r = 0.25 (<0.001). - PTA length, cognition, consciousness - employment at 2 years - emotional well being - FIMm with nursing contact time | FIM total ES= 0.46–0.72  FIM Cognitive ES = 0.35-0.43  SRM 0.53  More responsive than BI (ES = 0.4 SRM 1.0, t 1.1z 1.1; variance ratio 0.39 F 19.40 p<0.001).  Less responsive than   - GAS - CMSA disability inventory | Floor and ceiling effect | [45, 51, 53, 54, 71, 83, 87, 88, 92-94, 98, 110, 133, 135, 142, 152, 160] |
|  |  |  |  |  | |  |  |  |  |  |  |  |
| Global Ax Scale  (Self-report measure of response to treatment) | - | - | - | - | | - | - | - | - | - | - | - |
|  |  |  |  |  | |  |  |  |  |  |  |  |
| GAS  (Self-report or obs perf measure of individual goal attainment) | Majority of goal areas matched necessary domains. | - | - | ICCA,k 0.478  r = 0.92 - 0.94 | | - | - | LOA: -1.52 +/- 24.54 | 2 mo GAS predicted final GAS 0.66  70% agreement between participant and significant other ratings.  Greater goal achievement was associated with;   - higher mobility (FIM motor r = 0.55) - less depression (r = 0.46) - better self-efficacy r = 0.46) 6 months post.   Measure of;   - Activity (observed) r = -0.0039 to 0.77 - perceived activity and participation r = 0.45 – -0.51 p<0.005 - global clinical impression (clinical judgement of efficacy) r = 0.81, - vocational independence and outcome r = -0.34 - -0.69) - MAS 0.35, GAS change score and reduction in spasticity rho 0.28 p 0.04.   No relationship with;   - LOS -0.13 | ES 0.90  SRM: 2.4 t value 10.0 z value 1.4  Pre T Score 36.9 (6.3) post Ix 52.8 (6.2) t = -9.65 p<0.01  More responsive than FIM, BI | - | [42, 44, 60, 103, 105, 116, 125, 126, 157] |
|  |  |  |  |  | |  |  |  |  |  |  |  |
| Klein-Bell ADL Scale  (Self-report measure of global ADL) | - | - | - | - | | - | - | - | - | - | - | - |
|  |  |  |  |  | |  |  |  |  |  |  |  |
| LASIS  (Self-report measure of upper limb activity – active and passive) | - | - | - | - | | - | - | - | - | - | - | - |
|  |  |  |  |  | |  |  |  |  |  |  |  |
| MAL  (Self-report measure of upper limb activity) | - | Items removed due to missing data; write on paper (48%), put on makeup / shaving cream (20%).  92% item-total correlations >0.5. | - | - | | - | - | MDC AOU 16.8%, QOM 15.3% | Measures of;   - Activity r = 0.61-0.82 (p<0.01) - Participation r = 0.23 (p<0.05) - Impairment r = -0.06 - 0.84 (p<0.01) | - | Nil floor/ceiling effect | [48, 64, 89, 159] |
|  |  |  |  |  | |  |  |  |  |  |  |  |
| MAL-5  (Self-report measure of upper limb activity) | - | - | - | - | | - | - | - | - | - | - | - |
|  |  |  |  |  | |  |  |  |  |  |  |  |
| MAL-28  (Self-report measure of upper limb activity) | - | - | α = 0.94, caregiver 0.95 | - | | - | Patient reported QOM/AOU ICC 0.82/0.79, caregiver reported QOM/AOU ICC 0.72/0.66. | - | Patient and participant reported QOM/AOU with;   - accelerometry r = 0.52/0.47, p<0.01, r = 0.61/0.57, p<0.01, - SIS hand function r = 0.72/0.68, p<0.01, 0.40/0.35, <0.01 | - | - | [159] |
|  |  |  |  |  | |  |  |  |  |  |  |  |
| MI  (Obs perf measure of impairment and mobility) | - | - | α = 0.968 | MI arm spearman rho = 0.88 | | - | - | - | Measures of;   - Activity r = 0.73–0.76, - Dexterity r = 0.36–0.53 - Global function r = 0.61–0.77 - Impairment r = 0.74–0.94,   Predictor of independence | ES 0.49 | Ceiling effect 18%  No floor effect | [41, 49, 99, 155, 164] |
|  |  |  |  |  | |  |  |  |  |  |  |  |
| NHPT  (Obs perf measure of dexterity) | - |  | - | Rho = .83–0.99 | | Rho = 0.68–0.90 | Rho = 0.75–0.99 | 20% score change indicates true change | Measures of;   - Activity r = 0.36–0.76 - Grip and dexterity r_s_ =0.61–0.95 - Self-reported hand use r_s_ = 0.53–0.66 - Global measures of function r = -0.19–0.61 - Disability r = 0.63 - Cognition r = -0.20 - -0.65 p<0.01 - HRQoL r = 0.08 - Age and impairments r = -0.05-0.48   9HPT more sensitive than Frenchay Arm Test,  Did not predict overall HRQoL r = -0.08 | ES = 0.52–0.66 | Floor effect = 75% (stroke)  Nil floor or ceiling (MS) | [39, 40, 80, 95, 99, 130, 140, 151] |
|  |  |  |  |  | |  |  |  |  |  |  |  |
| OHS  (Self-report measure of global ADL) | - | - | - | - | | - | - | - | Not predictive of caregiver burden (Relatives Stress Scale P<0.0001; Bakas Caregiver Outcomes Scale P 0.059).  Predicted number of services required (13% of variance F=9.53 d.f.1=1, d.f.2=62 P=0.001, 14%) and amount of time provided (26.5% variance F=26.39, d.f.1=1, d.f.2=64 P<0.001 and 28.2%). | - | - | [145, 153] |
|  |  |  |  |  | |  |  |  |  |  |  |  |
| PDS/CBS  (Self-report measure of upper limb activity – active and passive) | - | - | - | - | | - | - | - | - | - | - | - |
|  |  |  |  |  | |  |  |  |  |  |  |  |
| SIS  (Self-report measure of global HRQoL) | - | V3 developed following deletion of 5 items secondary to misfit to the construct.  Unidimensional domains, ranging in item difficulty and able to discriminate.  8 domains can form single index (α = 0.93, accounting for 68.76% of variance) | α = 0.86 – 0.89 excluding emotion domain, 0.76 – 0.83 | Self and proxy agreement ICC = 0.50-0.83. | | - | ICC = 0.62-0.94 (Mail)  ICC = 0.91-0.98 (telephone) | - | Measures of;   - Activity (patient reported) r = 0.42-0.77, (proxy reported) r = 0.37–0.78 - Global measures of function r = 0.40-0.98   Perceived recovery predicted perceived participation.  Discriminant validity;   - Stroke (mean 9 points lower) - Disability (Kruskal-Wallis test 0.0002 – 0.0694)   Telephone administration;   - FIM and SF-36 r = 0.362-0.858 | - | No evidence of floor or ceiling effect (complete SIS)  Ceiling effect 32.2% (participation domain) | [65-67, 72, 100, 111, 113, 171] |
|  |  |  |  |  | |  |  |  |  |  |  |  |
| SA-SIP30  (Self-report measure of global HRQoL) | - | - | - | - | | - | - | - | Greater disability (DAS) associated with higher SA-SIP30 (P<.05)  6 mo SA-SIP30 was not predicted by admission NIHSS r = 0.11  Accounted for 53% of variance in predicting Reintegration to Normal Living R2 =0.63, P<0.0001). | - | - | [59, 70] |
|  |  |  |  |  | |  |  |  |  |  |  |  |
| SF-36  (Self-report measure of global HRQoL) | - | Inconsistent support for two factor structure of scale in TBI  Scaling assumptions were not fully satisfied for scale or summary scores in stroke. | α >0.7 to 0.96 for domains excluding general health and vitality | - | | - | Self-reported ICC = 0.30 to 0.96,  Proxy ICC = 0.24 to 0.76,  Self and proxy ICC = 0.28 to 0.80 | - | Physical and mental construct validity supported – limitations in social functioning  Measures of;   - Global measures of function r = 0.16–0.69 - Disability (F = 19.7 – 48.8, df = 5, all p<10) - positive correlation with work and study - Depression r = >0.50 - Cognition <0.50 - no correlation with age - fatigue r = -0.31- -0.72 - summary scores had weak to no association with patients rating of severity of symptoms and quality of life (r = 0.06 - -0.69)   More sensitive than BI  Discriminates between TBI, MS and stroke severity  Inconsistent discrimination in TBI severity using summary scores | Dimensions ES = 0.01–0.30  SRM = 0.39 – 0.02 (1-3mo)  SRM = -0.15 to 0.88 (3 -6mo)  Less responsive than FIM | Stroke  (< 6 mo) floor effect = 23 – 85%, ceiling effect = 16 – 54%  Stroke (> 6mo) Floor effect = 17 – 61%), Ceiling effect = 16 - 52%).  MS  Floor effect = 21 – 85%  Ceiling effect = 18 – 74%  TBI Floor effect = 44 - 57% Ceiling effect = 17 – 38%  MID: Physical functioning 4-9, role physical 6-8, social functioning 6-7, PCS 6 points. | [30, 61, 62, 68, 75, 77, 78, 85, 86, 96, 97, 122, 123, 128, 131, 134, 139, 144, 146, 149, 162, 163, 169] |
|  |  |  |  |  | |  |  |  |  |  |  |  |
| UL-MAS  (Obs perf measure of upper limb activity) | - | Unidimensional scale  Inconsistencies within hierarchical scoring of items  Wrist radial deviation in > 65 years not indicative of motor function | Summed α = 0.83  Item 6 0.893, item 7 0.889, item 8 0.854 | % agreement mean 88 – 95 (40 - 100%)  Kappa 0.93 – 1.0, mean % agreement 88-95 | | Kendall’s (Tau) 0.74 -0 1.00 | - | - | predictive and discriminant validity within UL function  Did not predict walking speed r = 0.06 – 0.09  Correlation with global measures of function <0.70  Rasch based more precise than summative scoring, admission (15% RP, 1.15; 95% CI: 1.01, 1.40) and discharge (11%, RP, 1.11; 95% CI: 1.02, 1.23) | - | Floor effect 0 – 38%.  Ceiling effect 0 – 67%. | [47, 101, 104, 110, 117, 119, 120, 127, 138, 147] |
