# Supplementary material for: Psychometric properties of measures of upper limb activity performance in adults with and without spasticity undergoing neurorehabilitation–A systematic review
Source: PLoS One. 2021 Feb 11;16(2):e0246288. doi: 10.1371/journal.pone.0246288 (PMC7877653; doi:10.1371/journal.pone.0246288)
Supplement: S4 Table — (DOCX) [file pone.0246288.s006.docx]

S4 Table. Terwee criteria for good measurement properties

| **Measurement property** | **Rating** | **Criteria** |
| --- | --- | --- |
| Structural validity | + | **CTT**  CFA: CFI or TLI or comparable measure > 0.95 OR RMSEA < 0.06 OR SRMR < 0.08a  **IRT/Rasch**  No violation of unidimensionalityb: CFI or TLI or comparable measure > 0.95 OR RMSEA < 0.06 OR SRMR < 0.08  *AND*  no violation of local independence: residual correlations among the items after controlling for the dominant factor < 0.20 OR Q3’s < 0.37  *AND*  no violation of monotonicity: adequate looking graphs OR item scalability > 0.30  *AND*  Adequate model fit IRT: χ2 > 0.001  Rasch: infit and outfit mean squares ≥ 0.5 and ≤ 1.5 OR Z-standardized values > −2 and < 2 |
|  | ? | CTT: not all information for ‘+’ reported IRT/Rasch: model fit not reported |
|  | - | Criteria for ‘+’ not met |
| Internal consistency | + | At least low evidencec for sufficient structural validityd AND Cronbach’s alpha(s) ≥ 0.70 for each unidimensional scale or subscalee |
|  | ? | Criteria for “At least low evidencec for sufficient structural validityd” not met |
|  | - | At least low evidencec for sufficient structural validityd AND Cronbach’s alpha(s) < 0.70 for each unidimensional scale or subscalee |
| Measurement error | + | SDC or LoA < MICd |
|  | ? | MIC not defined |
|  | - | SDC or LoA > MICd |
| Hypotheses testing for construct validity | + | The result is in accordance with the hypothesisf |
|  | ? | No hypothesis defined (by the review team) |
|  | - | The result is not in accordance with the hypothesisf |
| Cross-cultural validity\measurement invariance | + | No important differences found between group factors (such as age, gender, language) in multiple group factor analysis OR no important DIF for group factors (McFadden’s R2 < 0.02) |
|  | ? | No multiple group factor analysis OR DIF analysis performed |
|  | - | Important differences between group factors OR DIF was found |
| Criterion validity | + | Correlation with gold standard ≥ 0.70 OR AUC ≥ 0.70 |
|  | ? | Not all information for ‘+’ reported |
|  | - | Correlation with gold standard < 0.70 OR AUC < 0.70 |
| Responsiveness | + | The result is in accordance with the hypothesisf OR AUC ≥ 0.70 |
|  | ? | No hypothesis defined (by the review team) |
|  | - | The result is not in accordance with the hypothesisf OR AUC < 0.70 |
| The criteria are based on, e.g., Terwee et al. [1] and Prinsen et al. [2] *AUC* area under the curve, *CFA* confirmatory factor analysis, *CFI* comparative fit index, *CTT* classical test theory, *DIF* differential item functioning, *ICC* intraclass correlation coefficient, *IRT* item response theory, *LoA* limits of agreement, *MIC* minimal important change, *RMSEA* root mean square error of approximation, *SEM* standard error of measurement, *SDC* smallest detectable change, *SRMR* standardized root mean residuals, *TLI* Tucker–Lewis index “+” = sufficient, “−” = insufficient, “?” = indeterminateaTo rate the quality of the summary score, the factor structures should be equal across studiesbUnidimensionality refers to a factor analysis per subscale, while structural validity refers to a factor analysis of a (multidimensional) patient- reported outcome measure cAs defined by grading the evidence according to the GRADE approach dThis evidence may come from different studies eThe criteria ‘Cronbach alpha < 0.95’ was deleted, as this is relevant in the development phase of a PROM and not when evaluating an existing PROM fThe results of all studies should be taken together and it should then be decided if 75% of the results are in accordance with the hypotheses | | |

| Criteria to quantify strength of relationships | |
| --- | --- |
| Effect size | Cohen’s d criteria: <0.5 for poor, 0.5-0.75 for moderate, 0.75-0.9 for good, and >0.90 for excellent. |
| Correlations | Cohen’s r criteria: 0.10 - <0.30 small, 0.30 - <0.50 medium, ≥0.50 large |

1. Terwee CB, Bot SD, de Boer MR, van der Windt DA, Knol DL, Dekker J, et al. Quality criteria were proposed for measurement properties of health status questionnaires. Journal of Clinical Epidemiology. 2007;60(1):34–42.
2. Prinsen CA, Vohra S, Rose MR, Boers M, Tugwell P, Clarke M, et al. How to select outcome measurement instruments for outcomes included in a “Core Outcome Set” - A practical guideline. Trials. 2016;17(1):449.
3. Cohen J. Statistical Power Analysis for the Behavioral Sciences, 2nd Edition. Routledge; 1988.
