# Supplementary material for: Psychometric properties of measures of upper limb activity performance in adults with and without spasticity undergoing neurorehabilitation–A systematic review
Source: PLoS One. 2021 Feb 11;16(2):e0246288. doi: 10.1371/journal.pone.0246288 (PMC7877653; doi:10.1371/journal.pone.0246288)
Supplement: Attachment — Submitted filename: Response to reviewers.docx [file pone.0246288.s007.docx]

Thursday 12^th^ November 2020

To the Editor in Chief,

On behalf of the authorship team, I am re submitting the manuscript entitled “Psychometric properties of measures of upper limb activity performance in adults with and without spasticity undergoing neurorehabilitation – A systematic review” for review.

Thank you for the opportunity to address your comments. We have considered and taken on board those comments and edited the manuscript accordingly. I have provided an itemised account in table format of all changes (see following page), in addition to tracked changes and accepted changes versions of the revised manuscript.

Thank you for your time and I look forward to your response.

Sincerely

Shannon Pike

Doctor of Philosophy Candidate

La Trobe University

| **Editor in Chief Comments** | **Author’s response** | **Changes to Text**  **(tracked version)** |
| --- | --- | --- |
| 1. Please ensure that your manuscript meets PLOS ONE's style requirements, including those for file naming. | Manuscript meets PLOS ONE’s style requirements |  |
| 2. If there are ethical or legal restrictions on sharing a de-identified data set, please explain them in detail (e.g., data contain potentially identifying or sensitive patient information) and who has imposed them (e.g., an ethics committee). Please also provide contact information for a data access committee, ethics committee, or other institutional body to which data requests may be sent. | All data is available within the submitted manuscript and supporting information. |  |
| **Reviewer 1 Comments** |  |  |
| This is a systematic review evaluates the quality of measures, and of related psychometric studies, assessing activity and/or participation in adults with upper limb spasticity. A second aim is to explore differences in the quality of the tools for adults with a neurological impairment but without upper limb spasticity.  The subject is interesting and deserves to be investigated. The procedure is well explained and the methodology used to determine the overall quality of the evidence (modified COSMIN GRADE) adequate. There are two major concerns which limit the interpretation of results:  -The second aim stated in the introduction (“differences in psychometric properties for the identified measurement tools for adults with a neurological impairment but without upper limb spasticity will be defined”) is not so clear. Plus, it is hard to reach considering the neurological conditions listed in the inclusion criteria in the Methods, as all these conditions are associated with spasticity (≥ 90% diagnosis of a following neurological condition; Stroke, Multiple Sclerosis, Cerebral Palsy, Traumatic Brain Injury, Anoxia). To define differences in psychometric properties for the identified measurement tools it would be better to include diseases that do not involve spasticity (e.g. Parkinson’s disease or neuromuscular disorders). | Thank you for your comment. The authors agree that the included neurological conditions are associated with upper limb spasticity and this was a deliberate inclusion. The prevalence and severity of upper limb spasticity for the included diagnoses and the impact on the use of the upper limb in everyday activity is highly variable. Due to this variation in severity and impact, the authors wanted to identify if the presence of upper limb spasticity impacted on which activity and participation measure should be selected based on psychometric evidence. I.e. does the presence of upper limb spasticity need to be considered when selecting an outcome measurement tool to evaluate activity and participation goals? This clinical goal led to the data analysis approach to synthesise all studies, and to then extract studies including participants identified to have upper limb spasticity to appraise the evidence separately.  Defining differences in the psychometric properties of the selected outcome measurement tools in diagnoses without spasticity to compare to those included here, as you recommend, is likely to be of clinical interest, but was outside the scope of this study. This study arose from wanting to understand which clinical tool to select when working with adults with neurological upper limb impairments that occur in those who suffer the impairment of spasticity, a goal met with our current paper. We have, however, clarified our aim further in the introduction. | Please see tracked changes in the introduction |
| -The rationale for inclusion of the 10 metre walk test is not clear. As the authors state, “the 10 metre walk test (10MWT) is a common measure of gait speed. Tools evaluating walking speed are relevant as involuntary and/or impaired arm movements can impact on balance and walking abilities when considering upper limb activity performance.”  Walking speed may correlate with arm movements in neurological diseases involving upper together with lower limbs. As the inclusion criteria did not require spare lower limb function such correlation may be found just because upper limb impairment is associated with lower limb impairment, which does not make the 10 meter test a good measure of upper limb function. The sentence should be modified and the need for including the 10 meter test revised critically and properly justified since the screening phase. | Thank you for this insightful comment which we believe will be clinically questioned as well – in our efforts for brevity we apologise that we were too brief on this measure. Unfortunately too, the Ashford & Turner-Stokes 2013 review does not explain the inclusion of the 10MWT, so we have sought to make their original rationale clearer in the present paper.  We have therefore revised this sentence now to read: “The 10 metre walk test (10MWT) is a common measure of gait speed. Tools evaluating gait speed are clinically used to measure the effect of upper limb spasticity on lower limb activity performance as involuntary and/or impaired arm movements may impact on balance and walking ability.”  Given our sampling frame for selection of assessments to include in this systematic review was drawn from the 2013 published systematic review of measurement tools reported in upper limb studies of spasticity management, and we published our protocol *a*priori, we respectfully cannot remove 10MWT from our screening. | Please see tracked changes line 724-727 |
| Minor:  -In “Now in 2019, such evidence is available” the year should be updated or removed. It is not clear why the availability in 2019 of a list of measures as in reference [6] (Ashford and Turner-Stokes, 2013) prompted a literature search up to 2016. It is like the study has been started in 2017, so “now in 2019” may be a little confusing. | Thank you for your recommendation. This section has been edited. | Please see tracked changes |
| The meaning of the sentence “a second reviewer screened a random 25% sample against inclusion criteria” should be better clarified as it is not clear what were the purpose and the result of 25% sample screening. | The authors have now edited the manuscript to increase clarity of this sentence. | Please see tracked changes |
| Reviewer 2  I would add a brief paragraph on the relevant literature summarizing findings from published systematic reviews on upper limb assessment tools. | Thank you for your comments, relevant findings related to psychometric evidence of tools from published systematic reviews have been added. | See tracked changes in the introduction. |
| The aim of this study was “to firstly critically appraise and summarize the quality of the psychometric properties of previously identified upper limb activity “. However authors included tools assessing other domains (e.g. the 10MWT). I agree that the 10MWT can be used as a proxy to assess ‘real-life’ activities however its inclusion in this paper reduces readability. I would recommend to remove these tools from the analyses.  Authors should also provide a rationale for the inclusion of tools measuring upper limb and activity and participation in real-life activities | See above. We respectfully cannot remove the 10MWT from our review given our published protocol and drawing the list of measures from an existing SR, however we have sought to add the clinical rationale for readers.  Thank you, we have now strengthened our introduction to provide greater detail. | Please see tracked changes |
| Methods. It is unclear why authors only selected tools published by Ashford and Turner-Stokes. This paper has been published in 2013. A wide range of new tools has potentially been excluded (e.g.Box and Block Test). Authors included the Arm Activity Measure (ArmA) as a newly developed measure. Following this reasoning other tools should be added.  Additionally, I do not understand why inclusion-exclusion criteria list “Undergoing rehabilitation”. I think this has greatly reduced number of collected papers. A clear example is the nine hole peg test. Authors reported less than 10 papers on psychometric properties of this tools that has been extensively studied in MS and other conditions. | Thank you for your comments. This systematic review aimed to firstly add to the evidence base and avoid duplication of existing knowledge and secondly to produce high-quality, timely evidence. There is an abundance of possible outcome measurement tools to select from, with a recent systematic review identifying 144 measures of outcome used in upper limb rehabilitation trials (Duncan Millar et al 2019). The authors therefore were required to devise scope for this review: with our very strong interest in adults with upper limb spasticity, we chose the latest review of upper limb outcome measures as the source of tools. The great limitation of the published reviews to date were the lack of COSMIN ratings – this is the unique and important data added by this paper which has taken some 4 years of analysis to produce (since each evaluation of each tool is a separate SR in its own right). At the time of registering our protocol, we therefore selected the most recent and comprehensive set of tools. We acknowledge that some tools had not been used in clinical trials or cohort studies of patients with spasticity, and therefore preferred assessments of readers may not appear in this extensive review– we have now made this clearer in our discussion.  Ashford and Turner-Stokes had already completed two systematic reviews (2008, 2013) to identify measures of ‘real-life’ upper limb activity. The authors did not believe there was merit duplicating the search to identify measures. To ensure currency, however, the authorship team did complete a review of clinical guidelines. This review of clinical practice guidelines on management of upper limb spasticity identified the ArmA as the only additional measure of upper limb activity not captured in the Ashford review. The Box and Block Test, for instance, was not identified in this process.  The authors acknowledge a risk of omission of outcome measurement tools and we have now made this clearer in the discussion. However, the Box and Block test as identified as an example by the reviewer was developed in 1957, therefore we maintain there has been sufficient time since release for this tool to have been identified within Ashford & Turner-Stokes review (which used a published search strategy). It was also not located in the Clinical Guideline search we conducted either as a recommended assessment, and therefore was not able to be included in our review.  ‘Undergoing rehabilitation’ did not exclude any studies and was not part of the search strategy therefore should not have contributed to the small numbers. Studies reporting on psychometric properties outside of a rehabilitation setting were excluded as participants did not meet diagnosis criteria, for example were healthy controls.  The PRISMA flow chart details exclusion reasons at full text. Studies were excluded at abstract screening stage due to being conducted in languages other than English or used as a comparator to validate anther tool, predefined criteria. COSMIN methodology details clear criteria for inclusion and exclusion of studies which were strictly adhered to so as achieve trustworthy results. | Please see tracked changes |
| Data Analysis Please add the Terwee’s quality criteria for measurement properties and provide a guide (categorization rules) to quantify the strength of the correlation (“very weak,” 0.20-0.39 “weak,”…). | Thankyou for the comment, Terwee’s quality criteria and Cohen’s correlation categorisation to quantify strength of correlations has been added as per recommendation. | Please see Supplementary file 6 |
| Results 95% of studies included post stroke participants. I think It is best to remove findings from conditions other than stroke. Likewise a very limited number of papers reported psychometric properties of several tools I think they can be removed from analysis.  In table 2 replace “While sample” with “Whole sample”, column name “Sample” can be misleading. It would also be useful adding an extra data on Minimal clinically important difference.  Overall I’m concerned about the number of studies included for each tool. For instance, authors reported 5 studies inquiring NHPT psychometric properties for stroke survivors. Similarly they reported only 5 studies including patients with multiple sclerosis. A review published in 2014 reported at least 14 papers investigating psychometric properties of this outcome measure in MS. | 97 (62%) of studies included only participants post-stroke. The remaining 38% of included studies included either participants with multiple sclerosis, post traumatic brain injury or a mixed sample including those diagnoses and stroke.  The authorship team are reluctant to remove the psychometric evidence drawn for the remaining studies due to the percentage but also due to compliance with the published study protocol.  When data was located regarding the minimal clinically important difference, this was reported and is presented in Supplementary Table 5 within the Interpretability column.  Strict adherence to the inclusion criteria resulted in study exclusion at both abstract and full text stages due to being administered in languages other than English. This was apparent for the NHPT. The PRISMA flow chart details exclusion reasons at full text. COSMIN methodology details clear criteria for inclusion of studies which was adhered to. | Line 219 has been edited due to an error in initially reported studies including participants post-stroke only. |
| Discussion should be better elaborated. Clinical implications should be included guiding clinicians in the choice of the best assessment tool. For example, coming back to the NHPT the most relevant problem in the clinical use of this tool for stroke survivors is the presence of floor effect. This makes NHPT a second line tool to assess upper limb in this condition. The reverse is true for ARAT in MS population. Comparisons between patient reported outcome and performance scale should also be considered. Finally, this paper reports findings on scales inquiring upper limb performances and scales on activity and participation the relationship between these two sets of instruments should be discussed. Are there activity and participation scales better representing upper limb impairments in real life activities than others? Are these scales better correlated with scales inquiring upper limb function. Are correlations with these scales better for Patients reported outcomes than functional scales? | Thankyou for your comments, we have now revised the discussion to make the clinical implications clearer.  We have also revised the discussion to make the point clearer that there are a wide range of upper limb assessment tools, and that each needs to be considered in the context of the service – however our findings do provide readers with a first-ever opportunity to compare the psychometric properties of a wide range of assessment tools which have been used with adults with upper limb spasticity in published trials, cohort studies and clinical practice guidelines. This level of information has not previously been made available to readers. | See tracked changes within the discussion |
