# Supplementary material for: Psychometric properties of measures of upper limb activity performance in adults with and without spasticity undergoing neurorehabilitation–A systematic review
Source: PLoS One. 2021 Feb 11;16(2):e0246288. doi: 10.1371/journal.pone.0246288 (PMC7877653; doi:10.1371/journal.pone.0246288)
Supplement: Attachment — Submitted filename: Response to Reviewers_11.1.2021.docx [file pone.0246288.s008.docx]

| **Editor in Chief Comments** | **Author’s response** |
| --- | --- |
| **Reviewer 1 Comments** |  |
| All comments have been addressed with the exception of the request on the timed walk test. Not only I had required to modify the sentence but also that the need for including the 10 meter test had to be revised critically and properly justified since the screening phase. I have also noticed that my fellow reviewer asked to address the issue of the 10 meter test. The indicated sentence has been modified, but it seems that no other action was taken in the paper towards the critical revision and proper justification, besides a response to reviewer "Given our sampling frame for selection of assessments to include in this systematic review was drawn from the 2013 published systematic review of measurement tools reported in upper limb studies of spasticity management, and we published our protocol apriori, we respectfully cannot remove 10MWT from our screening". | The authorship team apologise the previous revisions did not meet the reviewer requirements- we had added a brief explanation for why we were adhering to original set of tools, but now see that the reviewers both prefer not to include a LL measure.  Therefore, we have now removed the 10MWT from the included outcome measurement tools, with reasoning for this exclusion stated in the Method section. |
| **Reviewer 2 Comments** |  |
| I have read all changes. It seems that authors did not change the manuscript according to reviewers’ suggestions. The 10MWT has not been removed from the paper. Authors have added this sentence “The 10 metre walk test .... on lower limb activity performance as involuntary and/or impaired arm movements may impact on balance and walking ability”. First, they did not provide references supporting this statement, second disentangling the effect of UL spasticity on gait using TMWT is, at least, challenging.  In addition authors did not removed or added other conditions I understand the protocol was developed years ago and the importance to comply with the published study protocol. However these two constrains made the paper less informative: Psychometric properties of measures in adults with and without spasticity are unclear, and lack of methodological consistence makes decisions between measures difficult. Additionally, several tools widely used to assesses UL are not reported especially for pathologies different from stroke. In my opinion these caveats make difficult to “compare the psychometric properties of a wide range of assessment tools and different pathologies”. | We have now removed the 10MWT from the included outcome measurement tools with reasoning for this exclusion stated in the Method section.  Thank you for understanding the importance of our compliance with the registered and published study protocol. We do wish to correct that, as outlined in our paper, the diagnoses included in our systematic review were neurological conditions (not limited only to “stroke”). The search terms are outlined in Table 1, and these included Stroke, Multiple Sclerosis, Cerebral Palsy, Traumatic Brain Injury, and/or Anoxia (hypoxia).  We appreciate that there are tools used outside of these populations not reported and that there are tools which have not been psychometrically studied in a neurological population such that the tools have thus not been able to be included. We did, however, seek to include any additional tools recommended in clinical practice guidelines (in addition to the tools identified in the Ashford review). These points are outlined in our method.  We additionally appreciate that it may have been the reviewer’s point that without source studies, it is difficult to meet our study aim. Therefore, we have now also added this point to the limitations discussion of our paper:  *“The limited psychometric testing of the tools that were included was a further limitation, making it difficult to compare the psychometric properties of tools across different pathologies. This may mean that the preferred assessments of a reader does not appear in this extensive review, and where included, it may have only been tested in a single diagnostic population.”* |
